# Is there a relationship between the first-day results of anti-VEGF therapy for macular edema due to vascular diseases and longterm outcomes?

**DOI:** 10.5935/0004-2749.2022-0228

**Published:** 2023-09-27

**Authors:** Betul Onal Gunay, Gurkan Erdogan, Irfan Akalin, Ahmet Kalkisim, Cenap Mahmut Esenulku, Murat Gunay

**Affiliations:** 1 Department of Ophthalmology, Trabzon Kanuni Training and Research Hospital, University of Health Sciences, Trabzon, Turkey; 2 Department of Ophthalmology, Istanbul Faculty of Medicine, Istanbul University Istanbul, Turkey; 3 Department of Ophthalmology, Faculty of Medicine, Karadeniz Technical University Trabzon, Turkey

**Keywords:** Macular edema, Diabetic retinopathy, Diabetes mellitus, Retinal vein occlusion, Vascular endothelial growth factor-A, Angiogenesis inhibitors, Treatment outcome, Edema macular, Retinopatia diabética, Diabetes mellitus, Oclusão da veia retiniana, Fator A de crescimento do endotélio vascular, Inibidores da angiogênese, Resultado do tratamento

## Abstract

**Purpose:**

To evaluate early changes after the first antivascular endothelial growth factor injection for macular edema secondary to diabetic retinopathy and retinal vein occlusion and the relationship between longterm outcomes.

**Methods:**

The study enrolled patients who received anti-vascular endothelial growth factor injections for treatment-naive macular edema due to retinal vein occlusion and diabetic retinopathy. The central macular thickness was measured at baseline, post-injection day 1, week 2, and month 1, and at the last visit using spectral-domain optical coherence tomography. A good response was defined as a central macular thickness reduction of ≥10% on post-injection day 1. Patients were reassessed at the last visit with regard to treatment response on post-injection day 1 based on the favorable anatomic outcome defined as a central macular thickness <350 µm.

**Results:**

In total, 26 (44.8%) patients had macular edema-retinal vein occlusion and 32 (55.2%) had macular edema-diabetic retinopathy. The mean follow-up time was 24.0 (SD 8.5) months. A statistically significant decrease in the central macular thickness was observed in both patients with macular edema-retinal vein occlusion and macular edema-diabetic retinopathy after antivascular endothelial growth factor injection therapy (p<0.001 for both). All patients with macular edema-retinal vein occlusion were good responders at post-injection day 1. All nongood responders at post-injection day 1 belong to the macular edema-diabetic retinopathy group (n=16.50%). The rate of hyperreflective spots was higher in nongood responders than in good responders of the macular edema-diabetic retinopathy group (p=0.03). Of 42 (2.4%) total good responders, one had a central macular thickness >350 µm, whereas 5 (31.2%) of 16 total nongood responders had a central macular thickness >350 µm at the last visit (p=0.003).

**Conclusion:**

The longterm anatomical outcomes of macular edema secondary to retinal vein occlusion and diabetic retinopathy may be predicted by treatment response 1 day after antivascular endothelial growth factor injection.

## Abstract (pt)

**Objetivo:**

Avaliar as alterações precoces após a primeira injeção de anticorpos antifator de crescimento endotelial vascular (anti-VEGF) em casos de edema macular secundário à retinopatia diabética e oclusão da veia da retina e a relação entre essas alterações e o resultado a longo prazo.

**Métodos:**

Foram incluídos no estudo pacientes que receberam uma injeção de antifator de crescimento endotelial vascular para edema macular, virgem de tratamento e devido à oclusão da veia retiniana ou a retinopatia diabética. A espessura macular central foi medida no início do tratamento e no 1º dia, 2ª semana e 1º mês após a injeção, bem como na última visita, através de tomografia de coerência óptica de domínio espectral. Definiu-se uma “boa resposta” como uma redução ≥10% na espessura macular central no 1º dia após a injeção. Os pacientes foram reavaliados na última visita com relação à resposta ao tratamento no 1º dia após a injeção, com base em um resultado anatômico favorável, definido como uma espessura macular central <350 µm.

**Resultado:**

Foram registrados 26 (44,8%) pacientes com edema macular e oclusão da veia da retina e 32 (55,2%) com edema macular e retinopatia diabética. O tempo médio de acompanhamento foi de 24,0 meses (desvio-padrão de 8,5 meses). Foi observada uma diminuição estatisticamente significativa da espessura macular central após o tratamento antifator de crescimento endotelial vascular tanto em pacientes com edema macular e oclusão da veia retiniana quanto naqueles com edema macular e retinopatia diabética (p<0,001 para ambos). Todos os pacientes com edema macular e oclusão da veia retiniana responderam bem no 1º dia pós-injeção. Todos os que responderam mal no 1º dia pós-injeção pertenciam ao grupo com edema macular e retinopatia diabética (n=16,50%). A presença de manchas hiperrefletivas foi maior nos pacientes que responderam mal do que naqueles que tiveram boa resposta no grupo com edema macular e retinopatia diabética (p=0,03). Um dos 42 (2,4%) pacientes com boa resposta total teve espessura macular central >350 um, enquanto 5 (31,2%) do total de 16 pacientes com resposta ruim apresentaram espessura macular central >350 µm na última visita (p=0,003).

**Conclusão:**

O resultado anatômico de longo prazo do edema macular secundário à oclusão da veia retiniana e à retinopatia diabética pode ser previsto pela resposta ao tratamento no 1º dia após a injeção de antifator de crescimento endotelial vascular.

## INTRODUCTION

Diabetic retinopathy (DR) and retinal vascular occlusion (RVO) are the two most common retinal vascular diseases that cause macular edema (ME) and visual impairment. They usually disrupt the inner blood-retinal barrier (BRB). The recent treatment approach in both conditions is primarily injections of intravitreal anti-vascular endothelial growth factor (VEGF), which stabilizes the BRB and improves abnormal permeability^(1)^. Previous studies have shown the efficacy of intravitreal anti-VEGF injection for ME secondary to retinal vascular diseases^(2,3)^. The early clinical response to anti-VEGF therapy is often assessed based on examinations conducted on post-injection month 1^(4)^. Studies have reported complications and intraocular pressure changes at the immediate and early periods following intravitreal anti-VEGF injection.^(5,6)^ Data on the importance of the anatomical response at the early period after anti-VEGF injection remains limited^(7)^.

Interestingly, a study reported significant association between 1 h and 1 month central macular thickness (CMT) changes after intravitreal bevacizumab injection in patients with ME secondary to RVO (ME-RVO) and DR (ME-DR). The authors have concluded that the 1-h CMT status can predict the condition of the CMT 1 month after bevacizumab therapy^(8)^. In view of the previous findings on the relationship between early and late responses to intravitreal anti-VEGF injection, this study aimed to investigate early changes after the first anti-VEGF injection for ME-RVO and ME-DR using spectral-domain optical coherence tomography (SD-OCT) and evaluate the relationship between early response and longterm outcomes.

## METHODS

After local institutional review board approval (No. 2021/93), a retrospective chart review of patients who were followed up and received intravitreal anti-VEGF injection for ME-RVO and ME-DR in the ophthalmology department of a university hospital between January 2018 and May 2019 was conducted. Informed consent was not obtained given the retrospective study design. The study followed the tenets laid out in the Declaration of Helsinki.

### Patient selection

This study included patients aged ≥18 years who received intravitreal aflibercept (IVA; Eylea, 2 mg, Bayer HealthCare, Berlin, Germany) or ranibizumab (IVR; Lucentis®, 0.5mg, Novartis Pharma, Basel, Switzerland) injection for ME-RVO or ME-DR.

### Exclusion criteria

The exclusion criteria were as follows: irregular follow-up during the study, treatment history with intra-vitreal injections other than anti-VEGF (e.g, steroids), presence of other ophthalmic pathologies (e.g., glaucoma, uveitis, and amblyopia), history of any intraocular surgery, history of an uncomplicated cataract surgery either within 6 months before anti-VEGF therapy or at anytime during follow-up, presence of vitreoretinal interface problem and active proliferative DR, eyes with media opacity reducing the quality of OCT images, and follow-up duration <12 months. If both eyes were eligible for inclusion, the first treated eye was selected as the study eye.

### Examination protocol

The demographic data of the patients including sex, age, disease type, phakic-pseudophakic status, and symptom onset (months) were recorded. The type of anti-VEGF agent administered, number of injections, and follow-up time were also noted. For the statistical analysis, the Snellen best-corrected visual acuity (BCVA) was converted to the logarithm of the minimum angle of resolution (logMAR).

After three loading doses of intravitreal anti-VEGF injections, patients were recommended monthly visits and received on-demand re-injections whenever signs of disease activity were detected. Full ophthalmological examination including BCVA, intraocular pressure measurement with Goldmann applanation tonometer, and SD-OCT (Spectralis OCT; Heidelberg Engineering, Heidelberg,Germany) were performed at control visits. On SD-CT examination, CMT values were analyzed based on the central 1-mm zone of the macular thickness map defined in the Early Treatment Diabetic Retinopathy Study. The device’s software was used to calculate the CMT as the distance between the vitreoretinal interface and the outer border of the retinal pigment epithelium. The presence of several OCT biomarkers (i.e. intraretinal cyst [IRC], hyperreflective spots [HRS], subretinal fluid [SRF], and cystoid degeneration) were noted at baseline.

Fluorescein angiographic examination was completed at baseline visits and then upon the discretion of the physician during follow-up. Patient data, including BCVA and CMT, were recorded before injection, at day 1, week 2, and month 1, and at the last visit following injection. BCVA was not assessed 1 day after injection.

Patients were evaluated on 1 day after intravitreal anti-VEGF injections according to treatment response. Good response was defined as a CMT reduction of ≥10% on SD-OCT. Patients were further divided into two groups based on treatment response on post-injection day 1 as good and nongood responders. At the last visit, patients were reassessed with regard to treatment response on post-injection day 1 based on the favorable anatomic outcome defined as a CMT <350 µm.

### Statistical analysis

Statistical analysis was performed using IBM SPSS for Windows version 22.0 (IBM Corp., Armonk, NY, USA). Study data were evaluated using descriptive statistical methods (mean, standard deviation, minimum, and maximum). The Shapiro-Wilk test was used to determine data normality. Data distribution was assessed with the parametric test. The general linear model repeated mea sure was used to compare the distribution of homogeneous data at baseline, day 1, week 2, and month 1 following injection, and last visit. Bonferroni correction was performed to adjust for pairwise comparisons. Student’s t-test was used in the binary comparison of quantitive data between groups. The chi-square test and Fisher’s exact test was used for the qualitative data analysis. P<0.05 was accepted as statistically significant.

## RESULTS

This study included 58 eyes of 58 patients (female, n=30; male, n=28) who met the eligibility criteria. The mean patient age (SD) was 62.3 (10.4) years. Twenty-six (44.8%) patients had ME-RVO, and 32 (55.2%) had ME-DR. The mean symptom onset time (SD) was 2.8 (2.4) months. The mean follow-up time (SD) and number of injections (SD) were 24.0 (8.5) months and 8.3 (4.7), respectively. IVA was administered in 31 (53.4%) eyes and IVR in 27 (46.6%) eyes. Table 1 presents the demographic and clinical data of the patients.

**Table 1:** Clinical characteristics of the patients

	ME-RVO (n=26)	ME-DR (n=32)	p-value
Age (years), mean (SD)	63.0 (8.6)	61.8 (11.8)	0.66^a^
Female/ Male	13/13	17/15	0.81^b^
Phakic/ Pseudophakic (N)	18/8	22/10	0.96^b^
Follow-up (months), mean (SD, range)	23.3 (7.9, 12-37)	24.5 (9.1, 12-37)	0.59^a^
Time of symptom onset (months), mean (SD)	2.8 (2.4)	3.5 (1.8)	0.18^a^
Number of injections, mean (SD, range)	8.9 (5.2, 3-20)	7.8 (4.3, 3-18)	0.36^a^
IVA/IVR, (N)	12/14	19/13	0.31^b^
Good responders on post-injection day 1 (+/−)	26/0	16/16	<0.001^b^

Student t test^a^, Chi-square test^b^, p<*0.05.*

ME-RVO= macular edema secondary to retinal vein occlusion; ME-DR= macular edema secondary to diabetic retinopathy; SD= standard deviation; IVA= intravitreal aflibercept; IVR= intravitreal ranibizumab.

Table 2 summarizes CMT and BCVA changes. The CMT showed significant improvement following injection (p<0.001). The mean CMT (SD) was 584.3 (191.1) µm at baseline, 437.3 (126.0) µm on day 1, 347.0 (91.9) µm on week 2, 346.3 (99.8) µm on month 1, and 288.7 (55.3) µm at the last visit after injection. The mean BCVA significantly improved following injection (p<0.001). The mean BCVA (SD) was 0.75 (0.54) at baseline, 0.50 (0.44) on week 2, 0.37 (0.32) on month 1, and 0.36 (0.33) at the last visit after injection p<0.001).

**Table 2:** Changes in CMT and BCVA

		ME-RVO group (n=26)	ME-DR group (n=32)	All patients (n=58)
CMT, µm, mean (SD)	Preinjection	638.6 (229.6)	540.3 (142.0)	584.3 (191.1)
	Postinjection day 1	418.6 (103.7)	452.6 (141.4)	437.3 (126.0)
	p^a^	<0.001	<0.001	<0.001
	Postinjection week 2	311.1 (49.0)	376.187 (107.9)	347.0 (91.9)
	Postinjection month 1	302.8 (59.7)	381.8 (112.1)	346.3 (99.8)
	Last visit	278.6 (36.2)	296.9 (66.4)	288.7 (55.3)
	p^b^	<0.001**	<0.001**	<0.001**
BCVA, logMAR, mean (SD)	Preinjection	0.91 (0.61)	0.61 (0.43)	0.75 (0.54)
	Postinjection week 2	0.62 (0.51)	0.40 (0.35)	0.50 (0.44)
	Postinjection month 1	0.49 (0.40)	0.27 (0.19)	0.37 (0.32)
	Last visit	0.41 (0.42)	0.32 (0.22)	0.36 (0.33)
	p^c^	<0.001**	<0.001**	<0.001**

p^a^= pairwise comparisons with bonferonni correction; comparison between preinjection and postinjection day 1.

p^b^= General linear model repeated measures; comparison of all visits p^c^= General linear model repeated measures; comparison of all visits.

BCVA= best-corrected visual acuity; CMT= central macular thickness; logMAR= logarithm of the minimum angle of resolution; ME-DR= macular edema secondary to diabetic retinopathy; ME-RVO= macular edema secondary to retinal vein occlusion; SD= standard deviation.

**Figure 1:**
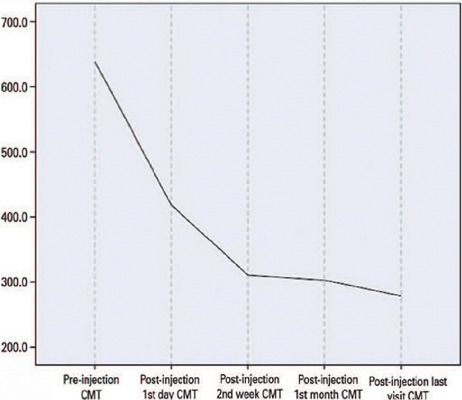
Changes in central macular thickness (CMT) trend.

**Figure 2:**
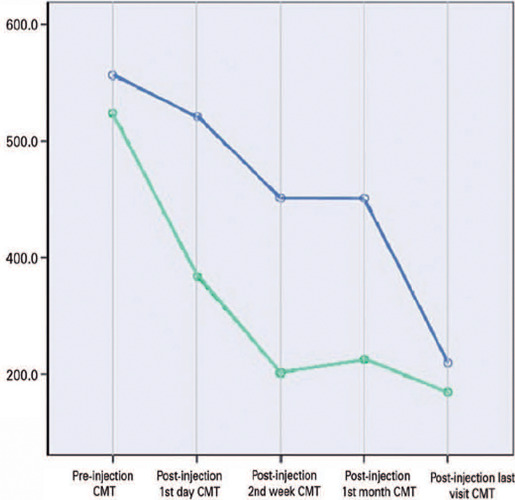
Changes in central macular thickness (CMT) trend during the study in good and nongood responders on post-injection day 1 (green and blue lines depict good and nongood responders, respectively, on post-injection day 1).

Compared with baseline, the amount of CMT reduction on post-injection day 1 was 34.4% (p<0.001) in the ME-RVO group and 16.2% (p<0.001) in the ME-DR group. Changes in the CMT trend are depicted in figures 1 and 2.

In this study, 42 (72.4%) patients (ME-RVO, n=26; ME-DR, n=16) had good response 1 day after injection. All patients with ME-RVO were good responders on post-injection day 1. These patients had a CMT <350 µm at the last visit (n=26, 100%). All nongood responders on post-injection day 1 belong to the ME-DR group (n=16, 50%). Moreover, 1 of 42 (2.4%) total good responders had a CMT >350 µm, whereas 5 (31.2%) of 16 total nongood responders had a CMT >350 µm at the last visit (p=0.003).

No difference was found in the mean number of injections (SD) during follow-up between good [8.1 (5.0)] and nongood [8.1 (4.6)] responders (p=0.67).

The mean symptom onset time (SD) was significantly shorter in good responders [2.9 (2.3) months] than in nongood responders [4.4 (1.3) months] (p=0.003). Furthermore, the mean symptom onset time was shorter in good responders [3.1 (2.0) months] than in nongood responders [4.4 (1.3) months] of the ME-DR group (p=0.04).

Table 3 shows the comparison between good and nongood responders regarding the presence of several OCT biomarkers (i.e, IRC, HRS, SRF, and cystoid degeneration) at baseline.

**Table 3:** Relationship between response status and SD-OCT biomarkers 1 day after intravitreal anti-VEGF injection in patients with ME-DR

	Good responders	Nongood responders	p-value
IRC (+)	16	13	0.22^a^
IRC (−)	0	3	
Cystoid degeneration (+)	2	2	0.70^a^
Cystoid degeneration (−)	14	14	
SRF (+)	6	9	0.28^b^
SRF (−)	10	7	
HRS (+)	9	15	0.03^a^
HRS (−)	7	1	

HRS= hyperreflective spots; IRC= intraretinal cyst; ME-DR= macular edema due to diabetic retinopathy; SD-OCT= spectral-domain optical coherence tomography; SRF= subretinal fluid; VEGF= vascular endothelial growth factor.

Fisher’s exact test^a^, Chi-square test^b^, p<0.05.

* Good response was defined as a reduction of CMT >10%.

As all patients with ME-RVO already had good response on post-injection day 1, only data from patients with ME-DR were included in the analysis to find out whether the presence of OCT biomarkers affect treatment response. The rate of HRS was significantly higher in nongood responders than in good responders 1 day after injection (p=0.03).

## DISCUSSION

Treatment response to intravitreal anti-VEGF injections for ME due to DR or RVO in the early period has not been extensively studied. Moreover, early treatment response has not been considered a surrogate marker to evaluate the longterm effectiveness of the treatment. In this study, we analyzed the early treatment response 1 day after intravitreal anti-VEGF injection using SD-OCT the ME-RVO and ME-DR groups. We further investigated the relationship between early treatment response and longterm treatment outcomes based on CMT changes. In our study, significant improvement was found on SD-OCT examinations in all patients 1 day after intravitreal anti-VEGF injections. The CMT significantly reduced in patients with RVO and DR. The CMT reduction rate was higher in patients with RVO than in those with DR on post-injection day 1. This early response gave us an idea about the relationship between the early effectiveness of the treatment and longterm treatment outcomes.

Patients who received intravitreal anti-VEGF therapy for ME due to RVO and DR were more likely to have improved visual acuity and reduced CMT^(9,10)^. Randomized controlled clinical trials have proved the efficacy of anti-VEGF agents in ME secondary to retinal vascular diseases ^(11,12)^. Although the duration of action of these drugs varied, the maximum effect on the tissue is often observed within month 1^(13,14)^. Similarly, in this study, anatomical improvements mostly occured during the first 2 weeks in patients with ME-RVO and ME-DR and continued to progress at similar levels until the end of month 1. Functional improvement also continued increa singly during the same period.

Regarding treatment response on post-injection day 1 after anti-VEGF therapy, all patients with ME-RVO (100%) were good responders following treatment where only 50% of patients with ME-DR were good responders to treatment based on the CMT reduction rate on SD-OCT analysis. A good response status significantly differed between the ME-RVO and ME-DR group on post-injection day 1. The CMT reduction rate 1 day after anti-VEGF injection were also higher in the ME-RVO group than in the ME-DR group. The incidence of non-good response was significantly higher in the ME-DR group because diabetes mellitus is a multifactorial, systemic disorder and the pathogenesis of diabetic macular edema (DME) is more complex^(15)^. Other possible reasons may be related to the fact that VEGF along with many other cytokines and/or pathways, low-grade inflammation, and neurodegeneration contribute to DME pathogenesis occurring over a longer period compared with ME secondary to RVO^(16,17)^. A study showed that microvascular changes occur before symptom onset in DME^(18)^. One may think that RVO is a more acute event in that patients are more likely to apply to the clinics earlier with shorter symptom onset time, which may affect the response status 1 day after anti-VEGF injection. Consistently, the symptom onset time was short in good responders than in nongood responders on 1 day after anti-VEGF injection. Therefore, early treatment with anti-VEGF injection may provide good response during the early post-injection period. Moreover, the different response status may be related to the VEGF load, which differs according to the disease types. Investigators have shown higher VEGF load in the anterior chamber and vitreous in the ME-RVO group than in the ME-DME^(19)^. Similarly, Nishinaka et al.^(20)^ concluded that the effect of anti-VEGF antibodies is generally dependent on intraocular VEGF levels; as the VEGF level increases, the anti-VEGF effect is seen even more.

To understand the relationship between the early response and longterm outcomes of anti-VEGF injection, we included patients who were regularly followed up with good adherence to intravitreal treatment. The number of good responders was significantly lower than that of nongood responders regarding having a CMT >350 µm at the last visit. Early changes on OCT after anti-VEGF injection might be an indicative data to determine longterm prognosis. We also did not identify an association between immediate treatment response and total number of injections.

Recently, interest in clinical evaluation of specific noninvasive prognostic biomarkers on OCT has increased. They also have begun to gain importance in the selection of agents^(21)^. Although two heterogeneous groups were analyzed in our study, all patients with RVO were good responders on post-injection day 1; thus, OCT biomarkers were evaluated only for patients with ME-DR. Nongood responders had significantly higher HRS incidence. This may support the idea that the absence of HRS may be an influential biomarker for anatomical improvement 1 day after anti-VEGF injection. The HRS in patients with ME-DR is usually defined as <30 µm diameter with similar reflectivity to the nerve fiber layer and the absence of back-shadowing^(22)^. Studies have proposed that HRS could be clusters of activated microglial cells and macrophages in response to an inflammatory process^(23)^ or lipid extravasations^(24)^ or migrating RPE cells^(25)^ or degenerated photoreceptor cells^(26)^. In patients with HRS, the predominance of other inflammatory cytokines besides VEGF may affect the immediate and late treatment response to anti-VEGF therapy. Some authors have even suggested that steroids should be the first-line therapy in the presence of HRS^(27)^.

The limitations of this study were its retrospective design, inclusion of a small number of patients, and absence of a control group. The use of two different anti-VEGF agents might also influence the study outcomes. However, we observed similar distribution pattern of anti-VEGF agent use in patients with ME-RVO and ME-DR.

In conclusion, we demonstrated significant anatomical changes on SD-OCT in both patients with ME-RVO and ME-DR 1 day after intravitreal anti-VEGF injection. These changes were more pronounced in ME-RVO cases. Based on our findings in this study, we believe that anatomical changes on SD-OCT 1 day after injection for ME in patients with RVO and DR may give an idea to predict the longterm anatomic outcomes.
